# Supplementary material for: Patterns of Intron Gain and Loss in Fungi
Source: PLoS Biol. 2004 Nov 30;2(12):e422. doi: 10.1371/journal.pbio.0020422 (PMC532390; doi:10.1371/journal.pbio.0020422)
Supplement: Table S1 — Also available at http://genes.mit.edu/NielsenEtAl/. (4.3 MB ZIP). [file pbio.0020422.st001.zip › NielsenEtAl/html/303.html]

AN3954.1.NCU03100.1.MG00175.1.FG01111.1


```
 CLUSTAL W (1.82) Multiple Sequence Alignments - Introns Inserted


Sequence 1: NCU03100.1	523 aa
Sequence 2: MG00175.1	524 aa
Sequence 3: FG01111.1	530 aa
Sequence 4: AN3954.1	489 aa
Alignment Length: 530 aa
Number Identitical Residues: 370 aa
Alignment Score (without introns) 16121


MG00175.1 	MSGAV2ARLAGLGRAFP------SMNAGSSSSSSTSNNADKSGS~ADLGLIGLAVM2GQN
NCU03100.1	MSGPV2ARLANIIVGG-------AVKPGSQSQSQSQTSANDAPS~ADIGLIGLAVM2GQN
FG01111.1 	MSGAV2ARLAHLNLGAPQQQHTSSTSPLSSNANQNATNADNAPS~ADLGLIGLAVM2GQN
AN3954.1  	-----~----------------------------------MTED2ADFGLIGLAVM~GQN
          	                                         : . **:******** ***

MG00175.1 	LILNMADHGFTICAFNRTVSKVDNFLANEAKG~KSIVGAHSTEEFVSKLKSPRRIMLLVQ
NCU03100.1	LILNMADHGFTVCAFNRTVSKVDHFLANEAKG~KSIVGAKSTEEFVSKLKSPRRIMLLVQ
FG01111.1 	LILNMADNGFTICAFNRTVSKVDRFLENEAKG~KSIVGAHSVEEFVSKLKSPRRIMLLVQ
AN3954.1  	LILNAADHGFTVCAYNRTTSKVDRFLENEAKG1KSIVGAHSVEEFCSKLKRPRRIMLLVM
          	**** **:***:**:***.****.** ***** ******:*.*** **** ******** 

MG00175.1 	AGKAVDDWIETLLPLLSKGDIIIDGGNSFFPDSNRRMKYLESKGLQFVGSGVSGGEEGAR
NCU03100.1	AGKPVDDWIETLLPLLEKGDIIIDGGNSHFPDSNRRTKYLASKGLHFVGSGVSGGEEGAR
FG01111.1 	AGKAVDEWIEKILPLLDAGDIIIDGGNSHFPDSNRRTKYLAEKSIRFVGSGVSGGEEGAR
AN3954.1  	AGNPVDQFIESLLPHLEEGDIIIDGGNSHFPDSNRRTKYLKEKGIRFVGSGVSGGEEGAR
          	**:.**::**.:** *. **********.******* *** .*.::**************

MG00175.1 	YGPSIMPGGSEAAWPHIKDIFQSISAKSDGEACCEWVGDEGAGHYVKMVHNGIEYGDMQL
NCU03100.1	YGPSLMPGGDEAAWPHIKDIFQSIAAKSDGEACCEWVGDEGAGHYVKMVHNGIEYGDMQL
FG01111.1 	YGPSIMPGGNEEAWPYIKDILQSISAKSDGDACCEWVGDEGAGHYVKMVHNGIEYGDMQL
AN3954.1  	YGPSLMPGGNEEAWPYIKDIFQSIAAKSDGEACCDWVGDEGAGHYVKMVHNGIEYGDMQL
          	****:****.* ***:****:***:*****:***:*************************

MG00175.1 	ICE0AYDIMKRGLGMSNKEIGDVFTQWNKGVLDSFLIEITRDIMYFNDEDGLPLVEKILD
NCU03100.1	ICE0AYDIMKRGLGLSHKEMGDVFAKWNKGVLDSFLIEITRDIMYFTDDDGTPLVEKILD
FG01111.1 	ICE0AYDIMKRGLGLSSKEIGDVFAEWNKGVLDSFLIEITRDIMYYNDDDDTALVEKILD
AN3954.1  	ICE0AYDILKRGVGLSSKEIADVFAKWNNGVLDSFLIEITRDILYFNDDDGTPMVEKILD
          	*** ****:***:*:* **:.***::**:**************:*:.*:*. .:******

MG00175.1 	KAGQKGTGKWTTINALDLGMPVTLIGEAVFGRCLSGIKEQRVRASQKLEYIGRTSSFEGN
NCU03100.1	KAGQKGTGKWTAVNALDLGMPVTLIAEAVLARCLSGIKEERVKASTKLEFVGRANKFEGD
FG01111.1 	KAGQKGTGKWTAVNALDLGMPVTLIAESVLSRCLSAIKDERATASTKLEFVSRTTKFEGD
AN3954.1  	KAGQKGTGKWTAVNALDLGMPVTLIGESVFARCLSALKDERIRASSLLN--GPTPEFTGD
          	***********::************.*:*:.****.:*::*  **  *:  . : .* *:

MG00175.1 	KEQFIKDLEQALYASKIISYAQGFMLMQE0AAKEYGWKLNKPSIALMWRGGCIIRSVFLK
NCU03100.1	KEQFLEDLEQALYASKIISYAQGFMLMQE0AAKEYNWKLNKPSIALMWRGGCIIRSVFLK
FG01111.1 	KKQFIDDLEQALYASKIISYAQGFMLMQE~AAREYGWKLNKPSIALMWRGGCIIRSVFLK
AN3954.1  	KEEFIADLEQALYASKIISYAQGFMLIQN0AAKEYGWKLNKPAIALMWRGGCIIRSVFLK
          	*::*: ********************:*: **:**.******:*****************

MG00175.1 	DITSAYRKNPDLENLLFDDFFNKAINNAQPGWREVVGRAAFLGIPTPAFSTALSWFDGYR
NCU03100.1	DITAAYRKNPDLENLLFDDFFNKAIHKAQPGWRDVVAKAALLGLPTPAFSTALSWFDGYR
FG01111.1 	DITHAYRSQPDLQNLLFDDFFNKAIHKAQPGWRDVIAKAALLGIPTPAFSTALSWFDGYR
AN3954.1  	DITEAYRKDPDLENLLFNDFFNKAIHNAQKGWRNVVSKGALWGIPTPAFSTALSFYDGYR
          	*** ***.:***:****:*******::** ***:*:.:.*: *:**********::****

MG00175.1 	TKDLPANLLQAQRDYFGAHTFRIKPEFANAKYPEGQDIHVNWTGRGGNVSASTYQA2
NCU03100.1	TKDLPANLLQAQRDYFGAHTFLVKPEYANEKYPEGKYIHVNWTGRGGNVSASTYQA~
FG01111.1 	TKDLPANLLQAQRDYFGAHTFRIKPEHASEKYPNGQDIHVNWTGRGGNVSASTYQA~
AN3954.1  	TKALPANLLQAQRDYFGAHTFRIKPEHASEKYPADKDIHVNWTGRGGQVSASTYTA~
          	** ****************** :***.*. *** .: **********:****** *
```
